# Risk and prognosis of secondary esophagus cancer after radiotherapy for breast cancer

**DOI:** 10.1038/s41598-023-30812-8

**Published:** 2023-03-09

**Authors:** Qianhui Sun, Yunru Chen, Tingting Li, Baoyi Ni, Xiaoyu Zhu, Bowen Xu, Jie Li

**Affiliations:** 1 Oncology Department, Guang’anmen Hospital, China Academy of Chinese Medical Sciences, No. 5, Beixian Pavilion, Xicheng District, Beijing, China; 2 Centre for Evidence-Based Chinese Medicine, Beijing University of Chinese Medicine, Beijing, China; 3 Beijing University of Chinese Medicine, Beijing, China

**Keywords:** Cancer, Oncology

## Abstract

Although radiation therapy (RT) improves locoregional recurrence and overall survival in breast cancer (BC), it is not yet clear whether RT affects the risk of patients with BC developing second esophageal cancer (SEC). We enrolled patients with BC as their first primary cancer from nine registries in the Surveillance, Epidemiology, and End Results (SEER) database between 1975 and 2018. Fine–Gray competing risk regressions were assessed to determine the cumulative incidence of SECs. The standardized incidence ratio (SIR) was used to compare the prevalence of SECs among BC survivors to that in the general population of the US. Kaplan–Meier survival analysis was applied to calculate the 10-year overall survival (OS) and cancer-specific survival (CSS) rates for SEC patients. Among the 523,502 BC patients considered herein, 255,135 were treated with surgery and RT, while 268,367 had surgery without radiotherapy. In a competing risk regression analysis, receiving RT was associated with a higher risk of developing an SEC in BC patients than that in the patients not receiving RT (*P* = .003). Compared to the general population of the US, the BC patients receiving RT showed a greater incidence of SEC (SIR, 1.52; 95% confidence interval [CI], 1.34–1.71, *P* < .05). The 10-year OS and CSS rates of SEC patients after RT were comparable to those of the SEC patients after no RT. Radiotherapy was related to an increased risk of developing SECs in patients with BC. Survival outcomes for patients who developed SEC after RT were similar to those after no RT.

## Introduction

Patients with cancer are typically treated by radiotherapy with curative intent. Radiation therapy (RT) remains a vital component of treatment and management of breast cancer (BC) at every stage, as it helps in reducing the risk of recurrence and death^1,2^. Oxygen-derived free radicals produced by high-dose ionizing radiation damage double deoxyribonucleic acid (DNA) and cause subsequent apoptosis of tumor cells^3,4^. Nevertheless, RT-related adverse effects such as lung and/or heart toxicity, cutaneous and subcutaneous fibrosis, rib fractures, and second primary cancers cannot be ignored^1,5^.

The risk of developing secondary malignancies is a form of late toxicity and mutagenic effect^6^. Up to 7.9% of cancer survivors in the US have multiple primary cancers (MPCs)^7^. Moreover, a substantial number of long-term survivors of BC are at risk of developing subsequent malignancies linked to late treatment outcomes^8^. The recurrence of esophageal cancer in BC survivors is of especial concern as the esophagus is located within or near the boundaries of several RT fields typically used for treatment, such as the internal mammary and supraclavicular lymph nodes^8,9^. In addition, since the mid-1980s, the use of breast-conserving therapies (lumpectomy and whole-breast radiotherapy) has been increasing, especially in patients with early invasive carcinoma and ductal carcinoma in situ for better outcomes, which may cause higher doses of esophageal radiation exposure than does mastectomy^10,11^. Previous studies on second cancers among BC survivors have reported a greater risk of developing esophageal cancer^10,12–16^, which is probably associated with RT^10,17–20^.

However, investigations into the risk of SECs(secondary esophageal cancer) following breast irradiation have yielded equivocal results^10,14,21^. A meta-analysis of data from 40,781 female patients of BC showed that RT is related to an increased risk of developing an SEC^22^. In contrast, other studies found that cancer incidence in women after breast irradiation did not differ considerably from that of the general population^13,23^. Owing to the rarity of SECs, further studies are needed to identify the relationship between breast RT and SECs. Theoretical considerations and modeling have shown that differences in the dose distribution of various RT techniques may contribute to different secondary cancer risks^24–26^. Unfortunately, there is a scarcity of large-sample, long-term follow-up clinical studies comparing the incidence of secondary malignancies across radiation modalities^27^.

Several large-scale studies have indicated that the clinical prognosis of BC survivors with MPC is not optimistic^28,29^. Etiologically, second radiation-induced cancers undergo DNA strand break repair and genomic impacts, which may alter the sensitivity to RT^30–32^ and lead to a different prognosis from the first tumors. A recent study revealed that for second radiation-induced tumors with specific genetic alterations, additional RT cannot prolong progression-free survival^33^. Despite this, few studies have investigated the survival rate of second radiation-induced malignancies, especially SECs. We herein aim to identify whether RT affects the risk of BC patients experiencing SECs and to investigate their long-term prognosis in a large population-based cohort study.

## Materials and methods

### Data source and study population

We retrieved custom data from the nine registries of the Surveillance, Epidemiology, and End Results (SEER) (with additional treatment fields) between January 1975 and December 2018. To distinguish between primary and recurring BC, the SEER program meticulously adheres to the 3rd International Classification of Diseases for Oncology (ICD-O-3) guidelines. Only patients with primary BC (C50.0–C50.9) at the localized or regional stage were enrolled. Patients who met the following criteria were excluded: (1) a diagnosis age of fewer than 20 years; (2) no cancer-directed surgery; (3) BC was not their first primary cancer; (4) survival less than one year after start of treatment; (5) distant metastases; and (6) missing or incomplete clinicopathological information.

Data on OPEC(only primary esophageal cancer) patients cohort were also obtained from the same database. OPEC patients had one and only one primary cancer, which was esophageal cancer.

### Interventions and outcomes

The SEER database comprises the initial treatment information. We placed patients with the first primary PBC into two groups: those who received RT and those who did not. The former (RT group) included BC patients who had surgery as well as (neo)adjuvant RT, whereas the latter no RT group included those who underwent surgery without radiotherapy.

The primary outcome of interest was the occurrence of an SEC, defined as a malignancy developing after at least one year of the initial therapy of BC patients. Follow-up ended at the point of interest, death, or the end of follow-up, whichever came first. The secondary outcomes were the overall survival (OS) and cancer-specific survival (CSS) of SECs. The endpoint events of OS and CSS were measured from the time after EC diagnosis. OS included all deaths from any cause during the follow-up period, while patients who were still alive were right-censored. CSS was defined based on the cause of death from esophageal etiology, whereas non-SEC deaths were considered competing risks, and patients alive were right-censored. Here the "non-SEC" developed a second cancer with a different cancer diagnosis.

### Statistical analysis

Differences in baseline patient, tumor, and treatment characteristics were assessed by the chi-square test or Mann–Whitney test, as applicable. Competing risk regression analysis (Fine–Gray model) was conducted to estimate subhazard ratios with their 95% confidence intervals (CIs) of risk factors for SEC occurrence after BC, assuming a non-SEC or all-cause death as competing risks^34^. Left truncation was used in this study, with age as the time scale. Left truncation time was one year after the diagnosis of BC. The univariate significant variables with *P*-value < 0.10 (two-sided) were entered in a multivariate model. Analyses were conducted using R software (version 4.1.0; R Foundation).

To quantify the risk of SECs in patients with BC in comparison to that in the general population of the US, we calculated the standardized incidence ratio (SIR) and 95% CIs by employing the Poisson distribution^34^, sex stratification, age at BC diagnosis, and calendar year of BC diagnosis. We defined SIR as the ratio of the observed incidence of SEC among BC patients to the incidence of EC in the general population of the US. SIR calculations were conducted by the MP–SIR session of SEER*Stat (version 8.3.9.2).

To minimize bias in survival comparisons and related confounding, 1:1 ratio propensity score matching (PSM) were applied to match SEC patients in the RT group and no RT(no radiotherapy) group. Next, SEC patients in the RT group and no RT group were matched with OPEC patients at a ratio of 1:5, respectively. Matching factors for PSM include age at EC diagnosis, year of EC diagnosis, tumor stage of EC, and treatment (surgery, radiotherapy, chemotherapy) for EC. Patients with OPEC were those who had been diagnosed with primary esophageal cancer and only had one primary malignancy during their follow-up. The OS and CSS curves were created by the Kaplan–Meier method. The log-rank test was employed to assess univariate Kaplan–Meier plots. A *P*-value of < 0.05 was considered statistically significant. These analyses were performed using R software (version 4.1.0). A list of abbreviations is showed in Table 1.

**Table 1:** A list of abbreviations.

Abbreviations	The full name
BC	Breast cancer
PBC	Primary breast cancer
SNBCs	Secondary non-breast cancers
SEC	Secondary esophageal cancer
non-SEC	Secondary non-esophageal cancer
OPEC	Only primary esophageal cancer
RT	Radiation therapy
no RT	No radiation therapy
IMRT	Intensity-modulated radiation treatment
UI	Upper-inner quadrant of breast
LI	Lower-inner quadrant of breast
UO	Upper-outer quadrant of breast
LO	Lower-outer quadrant of breast
CEN	Central portion of breast, nipple
OS	Overall survival
CSS	Cancer-specific survival
SIR	Standardized incidence ratio
PSM	Propensity score matching
HR	Hazard ratio
sHR	Subdistribution hazard ratio
Cl	Confidence interval
US	United States

### Ethical approval

The authors are accountable for all aspects of the work in ensuring that questions related to the accuracy or integrity of any part of the work are appropriately investigated and resolved. The study was conducted in accordance with the Declaration of Helsinki (as revised in 2013). The study were considered exempt by the institutional review board of Guang’anmen Hospital, China Academy of Chinese Medical Sciences, because the SEER data contain deidentified information.

## Results

### Baseline clinical characteristics

In total, 523,502 patients met the screening criteria. A total of 255,135 patients (48.7%) received surgery and (neo)adjuvant RT for a PBC, while 268,367 (51.3%) received surgery without radiotherapy. Primary cancers were located in the upper inner quadrant of the breast (UI, 10.6%), the lower inner quadrant of the breast (LI, 5.2%), the upper outer quadrant of the breast (UO, 35.6%), the lower outer quadrant of the breast (LO, 7.0%), the central portion quadrant of the breast (CEN, 6.5%), and other (axillary tail of breast and overlapping lesion of breast, 35.1%).

The total number of OPEC patients was 49,550, with the highest proportion of patients aged over 70 (43.7%) and white people (79.9%) accounting for more than half. After a minimum latency of one year from the initial RT, 433 patients were reported to develop an SEC: 207 in the RT group and 226 in the no RT group. The median follow-up time was 111 (55–90) months in the RT group and 123 (59–223) months in the no RT group. Compared with the patients in the no RT group, those in the RT group were younger and more recently diagnosed. In addition, the RT group comprised a lower proportion of patients belonging to white race, higher proportion of well or moderate differentiation, localized stage, UO site, and ductal tumors with *P* < 0.001. Fewer patients in the RT group received chemotherapy than those in the no RT group, with *P* < 0.001. The baseline characteristics of patients with BC who developed an SEC varied from those who did not. Only the year of BC diagnosis, tumor grade, and treatment were significantly different between the RT-SEC and (no RT)-SEC groups. Table 2 summarizes the baseline characteristics of BC patients who developed an SEC and those who did not.

**Table 2:** Comparison of baseline characteristics of breast cancer (BC) patients and secondary esophageal cancer (SEC) patients receiving radiotherapy (RT) or not.

Characteristic	All BC patients		*P*-value	All SEC patients		*P*-value
	Surgery without RT (n = 268,367)	Surgery with RT (n = 255,135)		Surgery without RT (n = 226)	Surgery with RT (n = 207)	
Median age at BC diagnosis, (IQR), years	62 (50–73)	59 (49–68)	** < 0.001**^**a**^	63 (55–71)	62 (54–70)	0.191^a^
Age at BC diagnosis, No. (%), years			** < 0.001**^**b**^			0.235^**b**^
20–49	63,763 (23.8)	65,487 (25.7)		23 (10.2)	31 (15.0)	
50–69	117,872 (43.9)	133,659 (52.4)		133 (58.8)	122 (58.9)	
≥ 70	86,732 (32.3)	55,989 (21.9)		70 (31.0)	54 (26.1)	
Median year of BC diagnosis (IQR)	1994 (1985–2005)	2004 (1996–2011)	** < 0.001**^**a**^	1989 (1982–1996)	1995 (1988–2002)	** < 0.001**^**a**^
Year of BC diagnosis, No. (%)			** < 0.001**^**b**^			** < 0.001**^**b**^
1975–1984	61,200 (22.8)	15,526 (6.1)		70 (31.0)	33 (15.9)	
1985–1994	76,391 (28.5)	37,005 (14.5)		91 (40.3)	66 (31.9)	
1995–2004	61,325 (22.9)	77,106 (30.2)		45 (19.9)	69 (33.3)	
≥ 2005	69,451 (25.9)	125,498 (49.2)		20 (8.8)	39 (18.8)	
Race, No. (%)			** < 0.001**^**b**^			0.226^**b**^
White	228,821 (85.3)	209,179 (82.0)		200 (88.5)	176 (85.0)	
Black	21,004 (7.8)	23,375 (9.2)		21 (9.3)	20 (9.7)	
Other	18,542 (6.9)	22,581 (8.9)		5 (2.2)	11 (5.3)	
Tumor grade, No. (%)			** < 0.001**^**b**^			** < 0.001**^**b**^
Grade I/II	104,186 (38.8)	143,618 (56.3)		72 (31.9)	100 (48.3)	
Grade III/IV	67,244 (25.1)	74,509 (29.2)		37 (16.4)	48 (23.2)	
Unknown	96,937 (36.1)	37,008 (14.5)		117 (51.8)	59 (28.5)	
Tumor stage, No. (%)			** < 0.001**^**b**^			0.630^**b**^
Localized	88,926 (33.1)	87,691 (34.4)		148 (65.5)	130 (62.8)	
Regional	179,441 (66.9)	167,444 (65.6)		78 (34.5)	77 (37.2)	
Tumor site, No. (%)			** < 0.001**^**b**^			0.179^**b**^
UI	24,596 (9.2)	30,861 (12.1)		23 (10.2)	32 (15.5)	
LI	12,709 (4.7)	14,726 (5.8)		15 (6.6)	14 (6.8)	
UO	90,797 (33.8)	95,472 (37.4)		81 (35.8)	83 (40.1)	
LO	18,445 (6.9)	18,386 (7.2)		15 (6.6)	17 (8.2)	
CEN	20,452 (7.6)	13,418 (5.3)		16 (7.1)	8 (3.9)	
Other	101,368 (37.8)	82,272 (32.2)		76 (33.6)	53 (25.6)	
Histology, No. (%)			** < 0.001**^**b**^			0.874^**b**^
Ductal	198,944 (74.1)	199,391 (78.2)		168 (74.3)	154 (74.4)	
Lobular	21,897 (8.2)	20,511 (8.0)		19 (8.4)	20 (9.7)	
Ductal and lobular	13,135 (4.9)	14,025 (5.5)		5 (2.2)	6 (2.9)	
Other	34,391 (12.8)	21,208 (8.3)		34 (15.0)	27 (13.0)	
Chemotherapy, No. (%)			** < 0.001**^**b**^			**0.019**^**b**^
Yes	204,518 (76.2)	151,600 (59.4)		39 (17.3)	56 (27.1)	
No/Unknown	63,849 (23.8)	103,535 (40.6)		187 (82.7)	151 (72.9)	

*P*-value was calculated using the Mann–Whitney U test (^a^) for continuous variables and x^2^ test (^b^) for categorical variables. *IQR* interquartile ratio; *BC* breast cancer; *SEC* secondary esophageal cancer; *UO* upper outer quadrant of breast; *UI* upper inner quadrant of breast; *LI*, lower inner quadrant of breast; *LO* lower outer quadrant of breast; *CEN* central portion quadrant of breast. The other sites of BC include axillary tail of breast and overlapping lesion of breast.

Significant values are in [bold].

### Cumulative incidence and SIR of SEC

**Figure 1:**
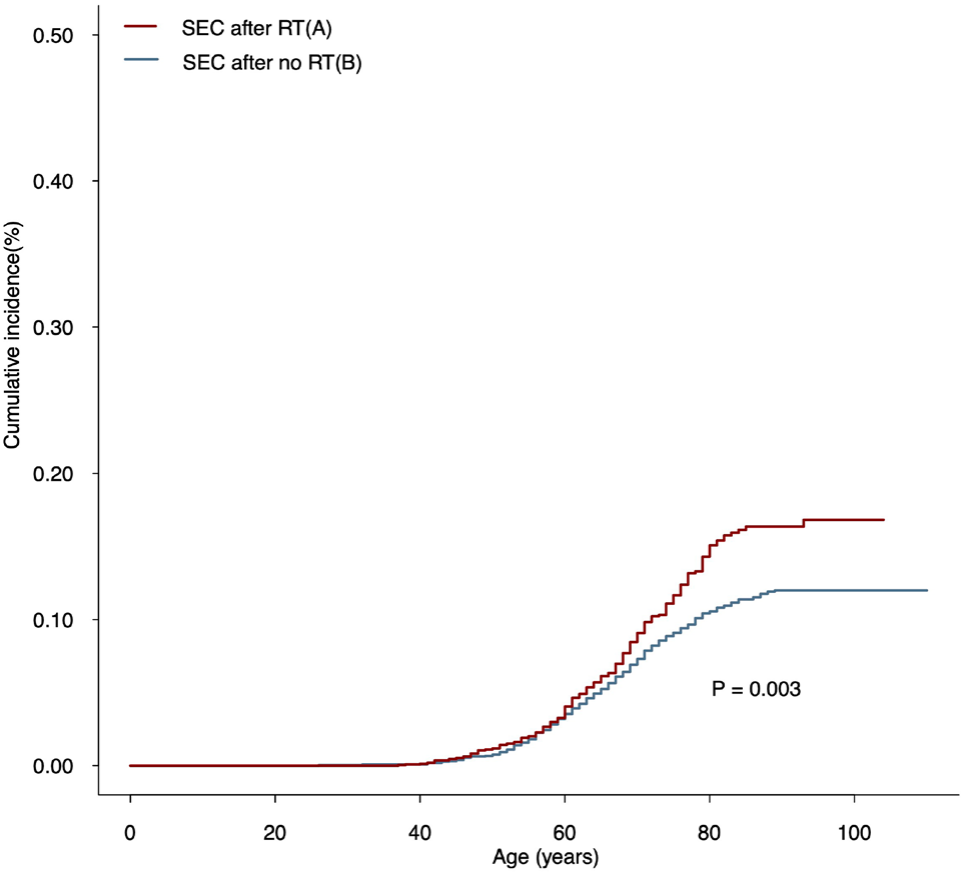
Comparisons of cumulative incidence of secondary esophageal cancer (SEC) between patients who received radiation therapy (RT) and patients who did not receive RT. *P* values were calculated with the Fine-Gray test. *RT* radiation therapy; *SEC* secondary esophageal cancer.

With a *P* of 0.003, SEC in BC survivors receiving RT had a greater 100-year cumulative incidence than that in BC survivors not receiving RT (Fig. 1). In addition, SIRs were computed to determine the incidence risk of SEC. Compared to the general population of the US, BC patients in the RT group were at a higher risk of developing SECs (SIR, 1.52; 95% CI, 1.34–1.71; *P* < 0.05; Supplementary Table 2); however, it was not observed for the patients in the no RT group. We next estimated the SIRs of SEC based on age range, latency from BC diagnosis, year of diagnosis, race, and tumor grade (Supplementary Table 2). In a dynamic diagnostic time–SIR plot, the SEC risk of RT remained steady at the lowest level among patients with BC diagnosed after around 2005 (Supplementary Fig. 2).

### Risk of developing SEC with RT

**Figure 2:**
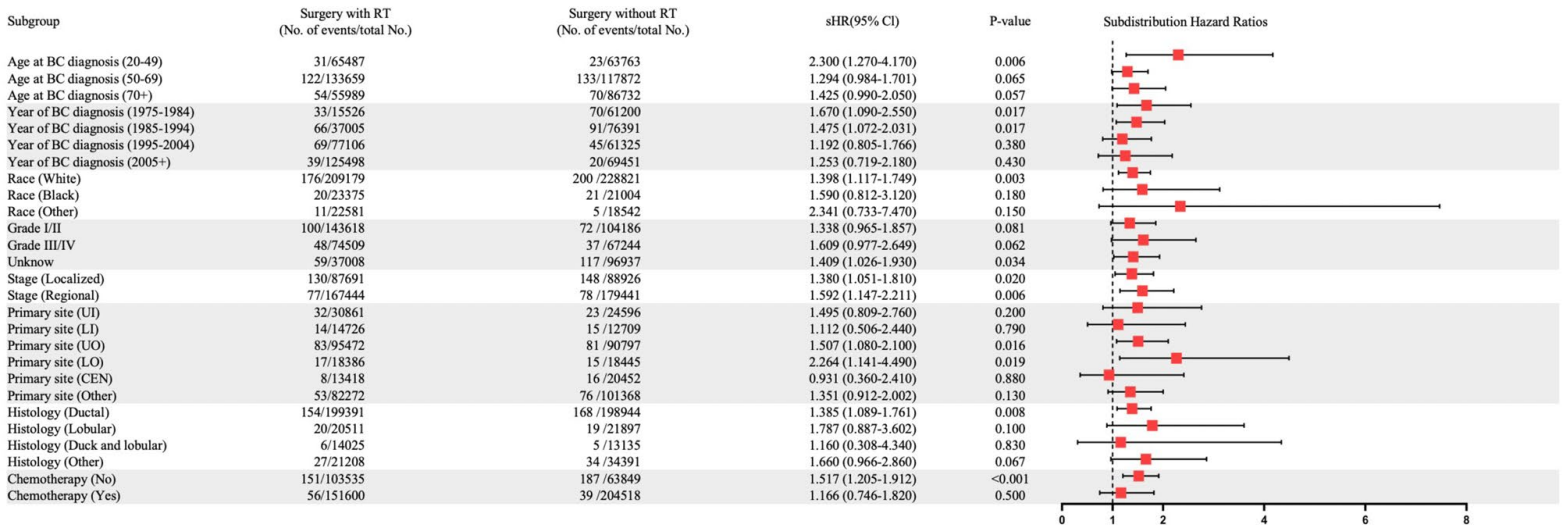
Subgroup analyses of competing risk regression for the risk of developing secondary esophageal cancer. *BC* breast cancer; *RT* radiation therapy; *UO* upper outer quadrant of breast; *UI* upper inner quadrant of breast; *LI* lower inner quadrant of breast; *LO* lower outer quadrant of breast; *CEN* central portion quadrant of breast. The other sites of BC include axillary tail of breast, overlapping lesion of breast. *sHR* subdistribution hazard ratio.

RT-related subdistribution hazard ratios in univariable competing risk regression was evaluated using the factors listed in Table 2. Univariate analysis showed that except for tumor stage and chemotherapy, the *P* values of other variables were less than 0.10. In multivariate analysis, characteristics such as age at BC diagnosis, year of BC diagnosis, latency, tumor site, and RT were associated with a greater risk of developing SECs (Table 3). In addition, RT was an independent risk factor for developing SECs in BC survivors (HR, 1.44; 95% CI, 1.168–1.774; adjusted *P* < 0.001). Subgroup studies were also conducted to assess the risk of developing SECs using competing risk regression. The higher risk of developing an SEC after RT was observed in almost all subgroups. However, not all subgroups had statistically significant results (Fig. 2).

**Table 3:** Univariable and multivariable competing risk regression analysis of risk of developing SEC in BC patients.

Characteristic	Univariable analysis		Multivariable analysis	
	sHR (95%Cl)	*P*-value	sHR (95%Cl)	*P*-value
Age at diagnosis	0.98(0.977–0.987)	** < 0.001**	0.98(0.979–0.990)	** < 0.001**
Year of diagnosis	0.97(0.964–0.979)	** < 0.001**	0.97(0.956–0.976)	** < 0.001**
Latency				
1–10 years	1		1	
11–20 years	1.35(1.099–1.660)	**0.004**	1.17(0.950–1.435)	0.140
> 20 years	1.22(0.935–1.600)	0.140	0.75(0.574–0.987)	**0.040**
Race				
White	1		1	
Black	1.21(0.877,1.670)	0.240	1.21(0.872–1.673)	0.250
Other	0.57(0.348–0.947)	**0.030**	0.62(0.374–1.025)	0.062
Tumor grade				
Grade I/II	1		1	
Grade III/IV	0.90(0.694–1.170)	0.430	0.76(0.582–1.000)	0.050
Unknown	1.47(1.186–1.820)	** < 0.001**	0.91(0.695–1.203)	0.520
Tumor stage				
Localized	1			
Regional	1.09(0.893–1.320)	0.410		
Tumor site				
UI	1		1	
LI	1.01(0.647–1.589)	0.950	1.02(0.651–1.601)	0.930
UO	0.86(0.636–1.172)	0.350	0.85(0.622–1.147)	0.280
LO	0.86(0.553–1.322)	0.480	0.85(0.546–1.308)	0.450
CEN	0.62(0.385–1.005)	**0.053**	0.64(0.394–1.026)	0.064
Other	0.67(0.491–0.922)	**0.014**	0.66(0.480–0.906)	**0.010**
Histology				
Ductal	1		1	
Lobular	1.05(0.765–1.469)	0.760	1.14(0.813–1.601)	0.450
Ductal and lobular	0.51(0.280–0.932)	**0.029**	0.57(0.312–1.039)	0.066
Other	1.20(0.912–1.578)	0.190	1.06(0.802–1.406)	0.680
Chemotherapy				
No/unknown	1			
Yes	0.92(0.736–1.150)	0.470		
Radiotherapy				
No/unknown	1		1	
Yes	1.20(0.992–1.450)	**0.061**	1.44 (1.168–1.774)	** < 0.001**

*sHR* hazard ratio; *CI* confidence interval; *BC* breast cancer; *SEC* secondary esophageal cancer; *UO* upper outer quadrant of breast; *UI* upper inner quadrant of breast; *LI*, lower inner quadrant of breast; *LO* lower outer quadrant of breast; *CEN* central portion quadrant of breast. The other sites of BC include axillary tail of breast, overlapping lesion of breast.

Significant values are in [bold].

### Survival outcome

**Figure 3:**
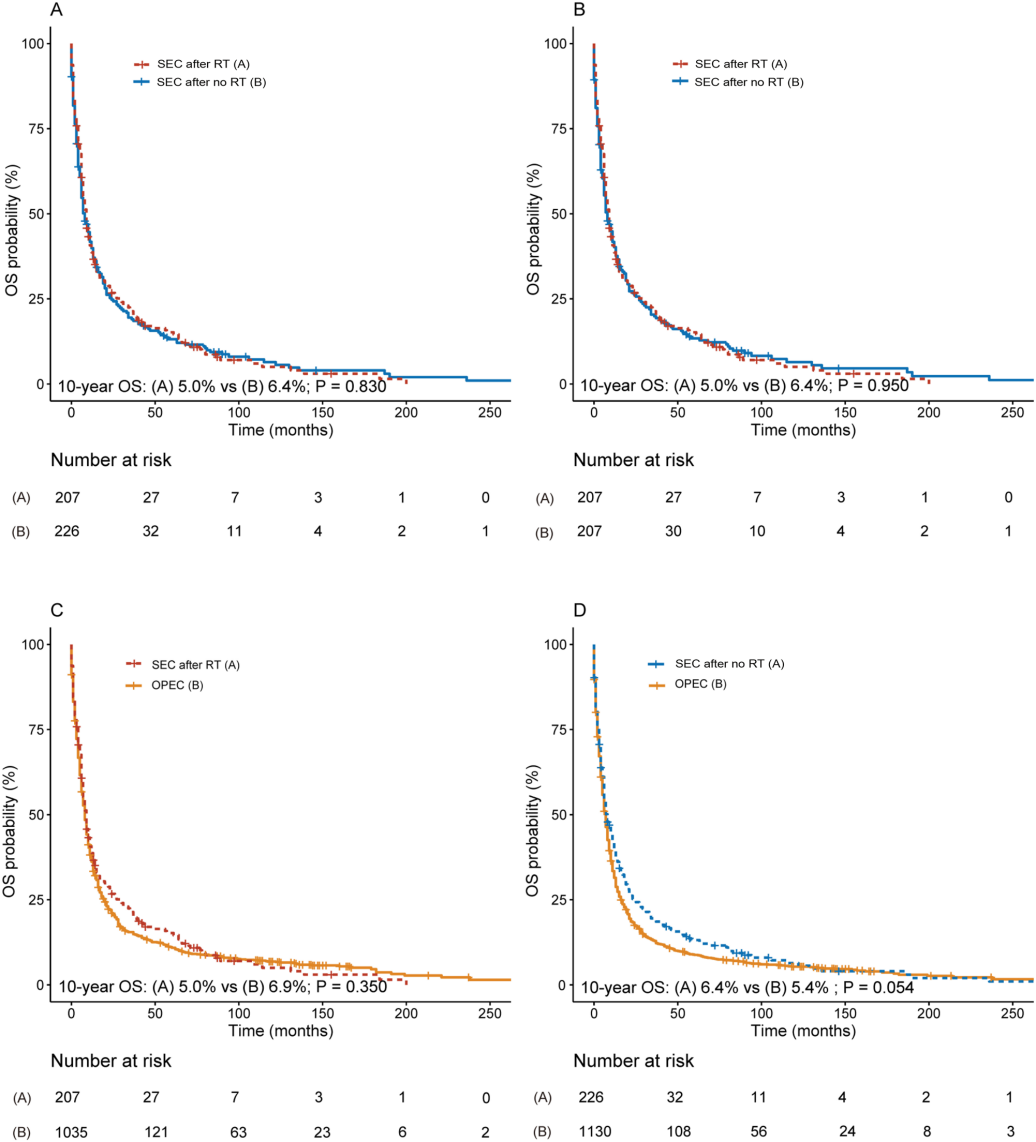
(**A**) Survival comparison between breast cancer (BC) patients who developed secondary esophageal cancer(SEC) after radiation therapy (RT) and BC patients who developed SEC after no RT (before PSM); (**B**) survival comparison between BC patients who developed SEC after RT and BC patients who developed BC after no RT(after PSM); (**C**) survival comparison between BC patients who developed SEC after RT and patients with only primary esophageal cancer (OPEC); (**D**) Survival comparison between BC patients who developed SEC after no RT and patients with OPEC. (**B**) PSM matched BC patients who developed SEC after RT and BC patients who developed SEC after no RT at a 1:1 ratio. SEC patients with RT (**C**) and without RT (**D**) were matched with OPEC patients based on propensity scores at a ratio of 1:5, and survival analysis was performed. The variables that were matched for PSM were age at SEC diagnosis, year of SEC diagnosis, race, SEC stage, and type of SEC treatment. The Supplementary Data reveal the complete patient characteristics of OPEC before and after PSM. *RT* radiation therapy; *no RT* no radiation therapy; *SEC* secondary esophageal cancer; *OPEC* only primary esophageal cancer; *OS* overall survival.

We conducted a comparison of survival rates of patients with SEC after RT and no RT. The baseline characteristics before and after propensity score matching are outlined in Supplementary Table 3 and Supplementary Table 4. We did not find any significant differences in the 10-year OS and 10-year CSS rates among patients developing an SEC after RT and those developing it after no RT, both before (10-year OS, 5.0% vs 6.4%; P = 0.830; 10-year CSS, 12.8% vs 18.1%, P = 0.800; Fig. 3A, Supplementary Fig. 1A) and after PSM (10-year OS, 5.0% vs 6.4%; P = 0.950; 10-year CSS, 12.8% vs 18.8%, P = 0.760; Fig. 3B, Supplementary Fig. 1B). PSM was applied to match the OPEC with the SEC to further analyze the survival outcomes of SEC.

Significant differences were observed in 10-year CSS between RT-SEC patients and matched OPEC patients (10-year CSS, 12.8% vs 11.3%, P = 0.027, Supplementary Fig. 1C). We also found that the 10-year CSS for (no RT)-SEC patients was considerably higher than that for comparable OPEC patients (10-year CSS, 18.1% vs 9.2%, P < 0.001, Supplementary Fig. 1D). Additionally, no significant difference was observed in 10-year OS between SEC patients with or without RT and matched OPEC patients, while SEC patients after no RT had a potential advantage in overall survival (RT-SEC: 10-year OS, 5.0% vs 6.9%, P = 0.350; (no RT)-SEC: 6.4% vs 5.4%, P = 0.054; Fig. 3C,D).

## Discussion

To the best of our knowledge, this is the first large population-based study to investigate the risk of developing SECs in BC survivors and to assess SEC survival outcomes. First, the cumulative incidence of SEC was greater in BC patients who underwent RT than in those that did not. RT was identified as an independent risk factor for the occurrence of SECs in BC patients. Second, the incidence of SECs in BC patients receiving RT was greater than that in the general population of the US. The risk of developing an SEC following RT decreased with latency and age. In the corresponding age groups of the general population in the United States, younger and middle-aged patients treated with RT are at greater risk than older patients. After around 1995, the SEC risk of RT stayed steady at its lowest level. In addition, no difference in the survival was observed between patients who received RT and those who did not.

Among the baseline characteristics listed in Table 2, the surgery with RT and surgery without RT groups showed significant differences in the year of diagnosis; the former includes more recently diagnosed patients. This may be because RT has become more common in the past 10 years. Consistent with other studies, we also identified RT as an independent risk factor for SEC occurrence in BC patients. Radiation has been reported to destroy DNA bases and induce strand breaks as well as inter-and intrastrand crosslinks^35^, which may also be the pathological mechanism of radiation-induced secondary esophageal malignancies. Moreover, postradiation cancers tend to cluster at the subtherapeutic outer edge, which may induce tumorigenesis^36^.

As indicated in the literature review, previous studies estimating the risk of developing an SEC in BC survivors have been controversial. These confusing results can be explained by the small sample size, length of follow-up duration, observation period selection, and study methodology. Taylor et al. performed patient data meta-analyses of 40,781 women randomly assigned to BC RT versus no RT in 75 trials to estimate the major absolute risks of second cancers. Their results revealed that modern RT has increased the incidence of developing SECs without prior BC recurrence^22^. However, the fact that only 33 SECs were observed and all involved trials were conducted before 2000, the small sample size of the population, and the latency period selected probably made the conclusion less convincing. Journy et al. conducted a nested case–control study within a cohort of 289,748 5-year cancer survivors from five countries treated with conventional or 3D conformal RT between 1943 and 2003. They reported similar results. Because of the much improved modern radiation techniques, the observation period selection for this may have limited statistical power^37^. As a reporting pooled study, the 2005 EBCTCG assessment of randomized studies that began in the mid-1970s and comprised just 31 SECs ultimately showed that annual SEC risk ratios for early BC were significantly increased^38^. Schaapveld et al. examined the risk of secondary non-breast cancers (SNBCs) in a recently treated population-based cohort of BC survivors based on the PALGA database. They observed that RT did not increase the risk of developing SEC^13^. The major strengths of our study are a long latency period, a large observation population, and a wide diagnosis year range to determine potential SEC risk using the SEER database.

Furthermore, discrepancies between previous studies can be attributed to differences in statistical methodology. In previous studies, four main statistical methods were used to quantify the risk of SEC following RT therapy for BC patients: logistic regression^8^, Poisson regression^17,21^, Cox regression^13,39^, and log-rank test^22,38^. However, the Fine–Gray competing risk model has not yet been utilized to assess the risk of SEC development until now. Fine–Gray competing risk analysis was performed to evaluate the risk of SECs, with the development of a non-SEC second main malignancy or all-cause mortality as the competing event. When the focus is on estimating incidence in the presence of competing risks, applying competing risk analysis may be a robust approach. But it should be noted that the results from Fine-Gray analyses cannot be readily compared to conventional survival analysis (i.e., cause-specific hazard regressions), as the resulting estimates are subdistribution hazard ratios, whereas the latter yields proper hazard ratios. All of this might add up to a more convincing reference for clinical prevention and therapy.

This study provides a novel evaluation of the dynamic incidence of SEC depending on age range, latency from BC diagnosis, and year of diagnosis. We discovered that after around 1995, the SEC risk of RT remained steady at the lowest level, presumably owing to modifications in irradiation methodology. In the 1990s, traditional two-dimensional (2D) radiotherapy progressed to three-dimensional (3D) conformal radiation treatment in broad, open fields. In the early 2000s, intensity-modulated radiation treatment (IMRT) was established, which demonstrated that the radiation beam could be more easily conformed to the target tumor^40^. Therefore, more precise radiotherapy target area formation may help in reducing the amount of radiation that the surrounding tissues and organs of a tumor are exposed to.

So far, there has been no study on the prognosis of RT-related SECs. This is a critical clinical concern for SEC since prognosis may be heterogeneous as different genetic signaling pathways may be induced after exposure to radiation. In our study, we did not find any difference in the survival rates among SECs after RT and no RT. We further conducted survival trials and evaluated the prognosis of SEC after RT or no RT with matched OPEC. Compared to OPEC, both RT-SEC and (no RT)-SEC exhibited a large CSS advantage; however, no OS. This result suggested that the initial therapeutic measures for BC may exert a positive impact on treatment outcomes following SEC diagnosis. Despite the differences in pathogenesis between SEC and OPEC, the former might still be sensitive to standard treatment for the latter.

This study has several limitations as well. First, potential biases may arise since the initial therapy for breast cancer is not randomized. Esophageal cancer may be caused by a combination of variables, including radiation exposure as well as the person's diet, cigarette smoking, alcohol exposure, family history, environmental factors, and other cancer-related therapies. Both initial cancer and the development of a second primary malignancy may have been influenced by unmeasured variables that cannot be involved in the SEER database. Although we used the competing risk model to adjust for confounding risk variables to avoid possible bias caused by a lack of randomization, these risk factors could not be balanced. Second, the limited information on radiation dosage, fractionation, and timing of radiation in the SEER database meant that we could not identify how esophageal volume, radiation dosage, fractionation, and timing of radiation affected the risk for SEC. Due to the limited treatment information in the SEER database, the no radiotherapy group included patients whose radiotherapy status was unknown. The SEER database includes the extent of surgery to be coded concerning the primary site; however, the specific type of surgery is not available in the SEER database, such as breast-conserving and mastectomy, which may affect the doses and range of radiation exposure. In addition, only the initial treatment information for BC is provided. It remains unclear whether the delayed RT is performed in subsequent treatments. Moreover, patients diagnosed with PBC in 2018 or later had a very short follow-up period of less than one year. This may lead to insufficient evidence to determine the occurrence of outcome events (whether SEC occurs in these PBC patients). As a result, the true risk of developing an SEC from RT might be underestimated.

## Conclusion

Radiotherapy was linked to an increased incidence of SEC in BC patients, suggesting that SEC surveillance in BC patients should be emphasized. If the typical symptoms of esophageal cancer, such as persistent retrosternal pain, backache, and choking sensation when swallowing, occur many years after RT for BC, endoscopic examination should be conducted in time to be alert to the possibility of developing SEC. More importantly, various methodologies were combined to obtain strong evidence of an increased risk of developing SEC in BC patients that received RT. The risk of developing an SEC was assessed based on several perspectives. As a result, a more valuable reference could be obtained for the treatment and follow-up of SECs in BC patients after RT.

## Supplementary Information

Supplementary Information.

## Data Availability

A population-based research was retrospectively operated with data from the SEER database, which incorporates national information on tumor samples from 18 large-scale cancer registries and is open to public for cancer studies (https://seer.cancer.gov/).
