# Supplementary material for: Risk and prognosis of secondary esophagus cancer after radiotherapy for breast cancer
Source: Sci Rep. 2023 Mar 9;13:3968. doi: 10.1038/s41598-023-30812-8 (PMC9998633; doi:10.1038/s41598-023-30812-8)
Supplement: Supplementary file 1 — Supplementary Information. [file 41598_2023_30812_MOESM1_ESM.pdf]

Supplementary Materials

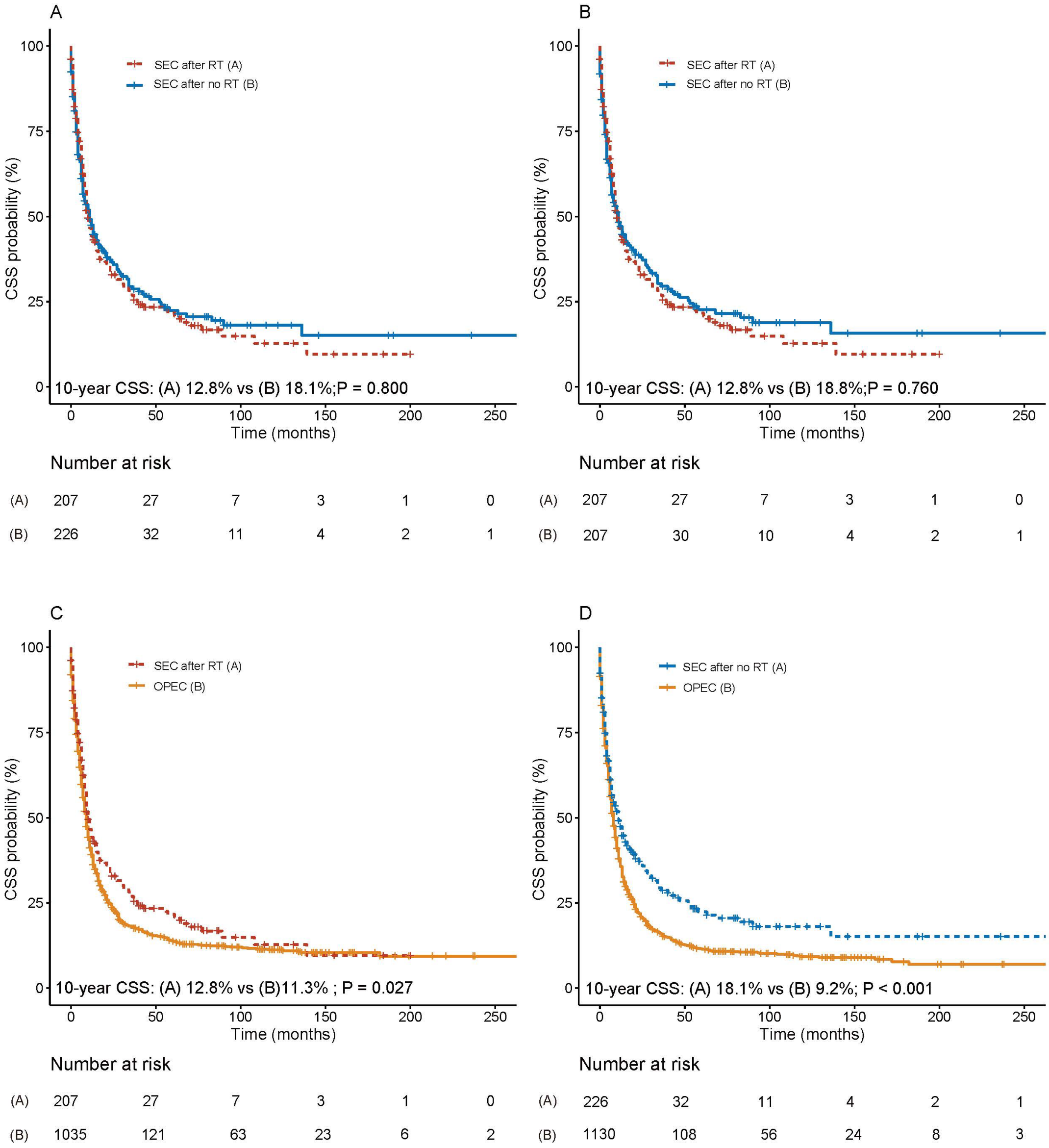

Supplementary Figure 1. (A) Survival comparison between breast cancer (BC) patients who developed secondary esophageal cancer(SEC) after radiation therapy (RT) and BC patients who developed SEC after no RT (NRT) (before PSM); (B) Survival comparison between BC patients who developed SEC after RT and BC patients who developed BC after NRT (after PSM); (C) Survival comparison between BC patients who developed SEC after RT and patients with only primary esophageal cancer (OPEC); (D) Survival comparison between BC patients who developed SEC after NRT and patients with OPEC.

NOTE (B) PSM matched BC patients who developed SEC after RT and BC patients who developed SEC after NRT at a 1:1 ratio. (C, D) are case-control comparisons of BC patients who developed SEC (cases) vs OPEC patients (controls), with a PSM ratio of 1:5 for SEC versus OPEC. The variables that were matched for PSM were age at SEC diagnosis, year of SEC diagnosis, race, SEC stage, and type of SEC treatment. The Supplementary Data reveal the complete patient characteristics of OPEC before and after PSM. HRs were calculated using Cox regression;

Abbreviations: HR, hazard ratio; BC, breast cancer; RT, radiation therapy; NRT, no radiation therapy;

CI, confidence interval.

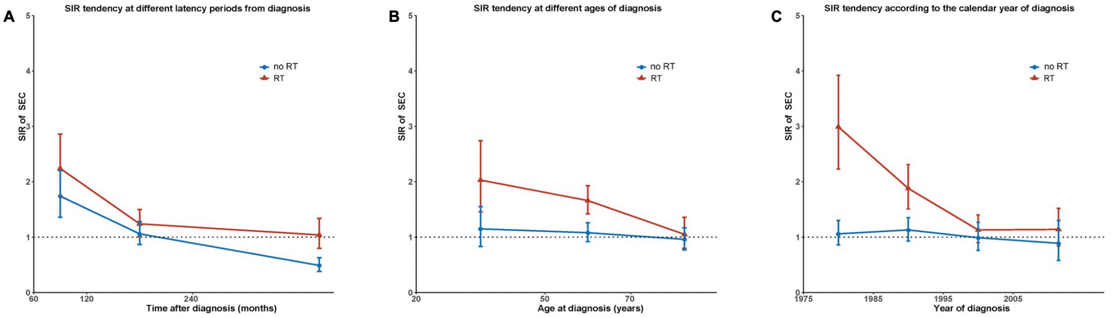

Supplementary Figure 2. (A) Dynamic standardized incidence ratio (SIR) for secondary esophageal cancer (SEC) in the latency-SIR plot; (B) Dynamic SIR for SEC in the age-SIR plot; (C) Dynamic SIR for SEC in diagnosis time-SIR plot. (A–C) SIRs of developing SEC in patients treated with radiation therapy (RT) versus the US general population are plotted, as well as patients treated without RT versus the US general population, and the incidence in the background US population is represented by the gray line (at y=1). The detailed data of SIRs are shown in Supplementary Table 2.

RT, radiation therapy; no RT, no radiation therapy; SIR, standardized incidence ratio; SEC, secondary esophageal cancer.

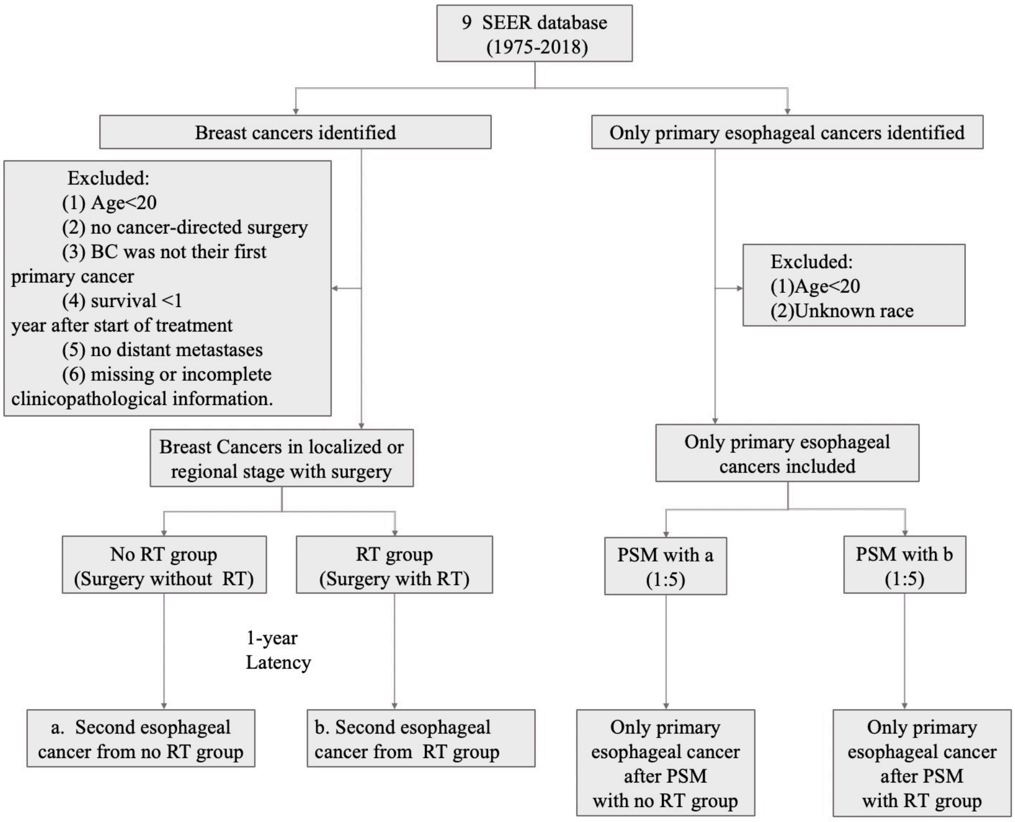

Supplementary Figure 3. A rough flow chart

Supplementary Table 1. Subgroup Analyses of Subdistribution Hazard Ratios of Developing Secondary Esophageal Cancer in BC Patients who Received RT versus Those who did not Receive RT.

**Subgroup**	**Surgery with RT (No. of events/total No.)**	**Surgery alone (No. of events/total No.)**	**sHR (95%CI)**	**P-value**
Age at BC diagnosis (20-49)	31/65487	23/63763	2.300 (1.270-4.170)	**0.006**
Age at BC diagnosis (50-69)	122/133659	133/117872	1.294 (0.984-1.701)	0.065
Age at BC diagnosis (70+)	54/55989	70/86732	1.425 (0.990-2.050)	0.057
Year of BC diagnosis (1975-1984)	33/15526	70/61200	1.670 (1.090-2.550)	**0.017**
Year of BC diagnosis (1985-1994)	66/37005	91/76391	1.475 (1.072-2.031)	**0.017**
Year of BC diagnosis (1995-2004)	69/77106	45/61325	1.192 (0.805-1.766)	0.380
Year of BC diagnosis (2005+)	39/125498	20/69451	1.253 (0.719-2.180)	0.430
Race (White)	176/209179	200 /228821	1.398 (1.117-1.749)	**0.003**
Race (Black)	20/23375	21 /21004	1.590 (0.812-3.120)	0.180
Race (Other)	11/22581	5 /18542	2.341 (0.733-7.470)	0.150
Grade I/II	100/143618	72 /104186	1.338 (0.965-1.857)	0.081
Grade III/IV	48/74509	37 /67244	1.609 (0.977-2.649)	0.062
Unknow	59/37008	117 /96937	1.409 (1.026-1.930)	**0.034**
Stage (Localized)	130/87691	148 /88926	1.380 (1.051-1.810)	**0.020**
Stage (Regional)	77/167444	78 /179441	1.592 (1.147-2.211)	**0.006**
Primary site（UI）	32/30861	23 /24596	1.495 (0.809-2.760)	0.200
Primary site（LI）	14/14726	15 /12709	1.112 (0.506-2.440)	0.790
Primary site（UO）	83/95472	81 /90797	1.507 (1.080-2.100)	**0.016**
Primary site（LO）	17/18386	15 /18445	2.264 (1.141-4.490)	**0.019**
Primary site（CEN）	8/13418	16 /20452	0.931 (0.360-2.410)	0.880
Primary site（Other）	53/82272	76 /101368	1.351 (0.912-2.002)	0.130
Histology (Ductal)	154/199391	168 /198944	1.385 (1.089-1.761)	**0.008**
Histology (Lobular)	20/20511	19 /21897	1.787 (0.887-3.602)	0.100
Histology (Duck and lobular)	6/14025	5 /13135	1.160 (0.308-4.340)	0.830
Histology (Other)	27/21208	34 /34391	1.660 (0.966-2.860)	0.067
Chemotherapy (No)	151/103535	187 /63849	1.517 (1.205-1.912)	**<0.001**

Chemotherapy (Yes)	56/151600	39 /204518	1.166 (0.746-1.820)	0.500

NOTE. Fine-Gray competing risk regression analyses are used to calculate the hazard ratios (HRs) and 95% confidence intervals (CIs) for SEC in breast cancer (BC) patients who received RT versus those who did not receive RT.

Abbreviations: HR, hazard ratio; CI, confidence interval; RT, radiation therapy; BC, breast cancer; EC, esophageal cancer; SEC, second esophageal cancer.

Supplementary Table 2. Standardized Incidence Ratio(SIR) of secondary esophageal cancer (SEC).

	RT			no RT		
	**Observed**	**SIR**	**95% CI**	**Observed**	**SIR**	**95% CI**
Total	270	1.52	1.34-1.71	300	1.05	0.93-1.18
Age at diagnosis, years						
20-49	42	2.03	1.46-2.74	43	1.15	0.83-1.55
50-69	171	1.66	1.42-1.93	164	1.08	0.92-1.26
70+	57	1.05	0.80-1.36	93	0.96	0.77-1.17
Latency, months						
12-59	41	4.67	3.35-6.33	57	3.99	3.02-5.16
60-119	66	2.24	1.74-2.86	71	1.74	1.36-2.20
120-239	101	1.24	1.01-1.50	108	1.06	0.87-1.28
240+	62	1.04	0.80-1.34	64	0.49	0.38-0.63
Year of diagnosis						
1975-1984	52	2.99	2.23-3.92	94	1.06	0.86-1.30
1985-1994	90	1.88	1.51-2.31	117	1.13	0.93-1.35
1995-2004	82	1.13	0.90-1.40	63	0.99	0.76-1.27
2005-2018	46	1.14	0.84-1.52	26	0.89	0.58-1.30
Race						
White	233	1.52	1.33-1.72	263	1.06	0.94-1.20
Black	23	1.39	0.88-2.08	27	0.99	0.65-1.44
Other	14	1.87	1.02-3.15	10	1.02	0.49-1.88
Tumor grade						
Grade I/II	122	1.30	1.08-1.55	87	0.94	0.76-1.16
Grade III/IV	61	1.48	1.13-1.90	52	0.94	0.70-1.23
Unknown	87	2.04	1.63-2.52	161	1.17	0.99-1.36

Standardized incidence ratios (SIR) were defined as the ratio of observed secondary esophageal cancer (SEC) cases among breast cancer (BC) survivors to the expected number of cases in the US general population and were stratified by age at BC diagnosis, latency period calendar, year of BC diagnosis, race, and tumor grade. Poisson exact methods were used to generate the 95% confidence intervals (Cl). RT, radiation therapy; no RT, no radiation therapy; SIR, standardized incidence ratios; Cl, confidence intervals.

Supplementary Table 3. Patients Characteristics of Second Esophageal Cancer Before and After PSM Matching.

Characteristic	Before PSM			After PSM		
	NRT(n=226)	RT(n=207)	P-value	NRT(n=207)	RT(n=207)	P-value
Age at EC diagnosis, No. (%), years			0.072			0.124
20-49	3 (1.3)	2 (1.0)		2 (1.0)	2 (1.0)	
50-69	70 (31.0)	86 (41.5)		66 (31.9)	86 (41.5)	
≥ 70	153 (67.7)	119 (57.5)		139 (67.1)	119 (57.5)	
Year of EC diagnosis, No. (%)			**<0.001**			**0.011**
1975-1984	15 (6.6)	2 (1.0)		4 (1.9)	2 (1.0)	
1985-1994	46 (20.4)	25 (12.1)		39 (18.8)	25 (12.1)	
1995-2004	77 (34.1)	60 (29.0)		77 (37.2)	60 (29.0)	
≥ 2005	88 (38.9)	120 (58.0)		87 (42.0)	120 (58.0)	
Race, No. (%)			0.226			0.299
White	200 (88.5)	176 (85.0)		183 (88.4)	176 (85.0)	
Black	21 (9.3)	20 (9.7)		19 (9.2)	20 (9.7)	
Other	5 (2.2)	11 (5.3)		5 (2.4)	11 (5.3)	
Tumor grade, No. (%)			0.510			0.590
Grade I	7 (3.1)	5 (2.4)		6 (2.9)	5 (2.4)	
Grade II	75 (33.2)	59 (28.5)		70 (33.8)	59 (28.5)	
Grade III	89 (39.4)	82 (39.6)		82 (39.6)	82 (39.6)	
Grade IV	7 (3.1)	4 (1.9)		5 (2.4)	4 (1.9)	
Unknow	48 (21.2)	57 (27.5)		44 (21.3)	57 (27.5)	
Tumor stage, No. (%)			**0.019**			**0.023**
Localized	75 (33.2)	58 (28.0)		69 (33.3)	58 (28.0)	
Regional	47 (20.8)	71 (34.3)		43 (20.8)	71 (34.3)	
Distant	58 (25.7)	44 (21.3)		53 (25.6)	44 (21.3)	
Unstaged	46 (20.4)	34 (16.4)		42 (20.3)	34 (16.4)	
Surgery, No. (%)			0.572			0.449
Yes	40 (17.7)	42 (20.3)		35 (16.9)	42 (20.3)	
No	186 (82.3)	165 (79.7)		172 (83.1)	165 (79.7)	
Chemotherapy, No. (%)			0.837			1.000
Yes	105 (46.5)	106 (51.2)		100 (48.3)	106 (51.2)	
No	121 (53.5)	101 (48.8)		107 (51.7)	101 (48.8)	
Radiation, No. (%)			0.373			0.623

Yes	131 (58.0)	117 (56.5)		116 (56.0)	117 (56.5)
No	95 (42.0)	90 (43.5)		91 (44.0)	90 (43.5)

NOTE. Breast cancer (BC) patients who developed second esophageal cancer (SEC) after radiation therapy (RT) were matched to patients after no RT(NRT), with a PSM ratio of 1:1. The variables matched for PSM included age at SEC diagnosis, year of SEC diagnosis, race, stage of SEC and type of treatment for SEC.

Abbreviations: BC, breast cancer; RT, radiation therapy; NRT, no radiation therapy; EC, esophageal cancer; SEC, secondary esophageal cancer.

Supplementary Table 4. Patients Characteristics of Secondary Esophageal Cancer and Matched Only Primary Esophageal Cancer.

Characteristic	**Surgery alone**			**Surgery with RT**		
	**Surgery alone (n=226)**	**PSM (n=1130)**	**P-value**	**Surgery with RT (n=207)**	**PSM (n=1035)**	**P-value**
Age at EC diagnosis, No. (%), years			1.000			1.000
20-49	3 (1.3)	15 (1.3)		2 (1.0)	10 (1.0)	
50-69	70 (31.0)	350 (31.0)		86 (41.5)	430 (41.5)	
≥ 70	153 (67.7)	765 (67.7)		119 (57.5)	595 (57.5)	
Year of EC diagnosis, No. (%)			1.000			1.000
1975-1984	15 (6.6)	75 (6.6)		2 (1.0)	10 (1.0)	
1985-1994	46 (20.4)	230 (20.4)		25 (12.1)	125 (12.1)	
1995-2004	77 (34.1)	385 (34.1)		60 (29.0)	300 (29.0)	
≥ 2005	88 (38.9)	440 (38.9)		120 (58.0)	600 (58.0)	
Race, No. (%)			**<0.001**			**0.001**
White	200 (88.5)	848 (75.0)		176 (85.0)	776 (75.0)	
Black	21 (9.3)	131 (11.6)		20 (9.7)	110 (10.6)	
Other	5 (2.2)	151 (13.4)		11 (5.3)	149 (14.4)	
Tumor grade, No. (%)			0.125			0.875
Grade I	7 (3.1)	46 (4.1)		5 (2.4)	39 (3.8)	
Grade II	75 (33.2)	324 (28.7)		59 (28.5)	310 (30.0)	
Grade III	89 (39.4)	404 (35.8)		82 (39.6)	394 (38.1)	
Grade IV	7 (3.1)	26 (2.3)		4 (1.9)	19 (1.8)	
Unknow	48 (21.2)	330 (29.2)		57 (27.5)	273 (26.4)	
Tumor stage, No. (%)			1.000			1.000
Localized	75 (33.2)	375 (33.2)		58 (28.0)	290 (28.0)	
Regional	47 (20.8)	235 (20.8)		71 (34.3)	355 (34.3)	
Distant	58 (25.7)	290 (25.7)		44 (21.3)	220 (21.3)	
Unstaged	46 (20.4)	230 (20.4)		34 (16.4)	170 (16.4)	

Surgery, No. (%)			1.000			1.000
Yes	40 (17.7)	200 (17.7)		42 (20.3)	210 (20.3)	
No	186 (82.3)	930 (82.3)		165 (79.7)	825 (79.7)	
Chemotherapy, No. (%)			1.000			1.000
Yes	105 (46.5)	525 (46.5)		106 (51.2)	530 (51.2)	
No	121 (53.5)	605 (53.5)		101 (48.8)	505 (48.8)	
Radiation, No. (%)			1.000			1.000
Yes	131 (58.0)	655 (58.0)		117(56.5)	585 (56.5)	
No	95 (42.0)	475 (42.0)		90 (43.5)	450 (43.5)	

NOTE. Breast cancer (BC) patients who developed second esophageal cancer (SEC) were matched with patients with only primary esophageal cancer (OPEC) at a PSM ratio of 1:5 for other SEC versus OPEC. The variables matched for PSM included age at SEC diagnosis, year of SEC diagnosis, race, stage of SEC and type of treatment for SEC.

Abbreviations: BC, breast cancer; RT, radiation therapy; NRT, no radiation therapy; EC, esophageal cancer; SEC, secondary esophageal cancer; OPEC, only primary esophageal cancer.
